# Comparative Anatomical and Morphometric Analysis of Eustachian Tube Across Species

**DOI:** 10.3390/audiolres15050141

**Published:** 2025-10-21

**Authors:** Rui Li, Yueqi Wang, Huaicun Liu, Xuan Fang, Quancheng Cheng, Man Li, Huiru Ding, Chao Wang, Ziyuan Wang, Baoshi Fan, Junxiao Jia, Yu Song, Zhen Zhong, Fei Shen, Weiguang Zhang, Junxiu Liu

**Affiliations:** 1 Department of Otolaryngology-Head and Neck Surgery, Peking University First Hospital, Beijing 100034, China; 2211110530@stu.pku.edu.cn (R.L.);; 2 Department of Human Anatomy and Histology and Embryology, School of Basic Medical Sciences, Peking University Health Science Center, Beijing 100191, China; 3 Laboratory Animal Center, Peking University Third Hospital, Beijing 100191, China

**Keywords:** eustachian tube, comparative anatomy, animal diseases, inflammation, NOX2

## Abstract

**Background/Objectives:** The Eustachian tube (ET) is a physiological channel connecting the middle ear with the external atmosphere. The ET plays a role in maintaining the pressure balance of the middle ear, protecting it from pathogen invasion, and cleaning secretions. Eustachian tube dysfunction (ETD) can lead to middle ear diseases in animals. The ET morphological structure are different across species. Therefore, we aim to compare the anatomical and morphological of ET across species. **Methods**: The combined skull base–nasal approach was used to anatomy ET. Hematoxylin-eosin, luxol fast blue myelin and immunohistochemical Staining were used to observe the morphology of ET. **Results**: There were significant differences in the size and structure of ET among species: the rodents ET (mouse: 1.152 ± 0.084 mm; rat: 3.738 ± 0.04355 mm) is characterized by cartilage and obvious bubbles; while the miniature pigs ET (32.34 ± 2.157 mm) has a chondroid conical structure similar to that of humans. ET inflammation model was built by intro-tympanic injection of lipopolysaccharide (LPS). NADPH oxidase 2 (NOX2) significantly increased by 38.6% in inflamed mice, causing ET oxidative stress. The expressions of inflammatory factors interleukin-1β (IL-1β) and cyclooxygenase-2 (COX2) increased by 28.4% and 30.8%, resulting in thickening of the ET mucosa and infiltration of inflammatory cells. **Conclusions**: The combined skull base–nasal approach was an effective method to anatomy ET across species. The morphology of ET varied across species and NOX2 might play an important role in ET inflammation.

## 1. Introduction

The Eustachian tube (ET) is the physiological channel connecting the middle ear with the external atmosphere. The incidence of Eustachian tube dysfunction (ETD) is 40% in children and 1–5% in adults [1,2], which is an important cause of protracted otitis media and hearing loss [3,4], and seriously affects the quality of life of patients [5]. The ET structure is the basis for maintaining ET function. The difficulty in obtaining human ET limited the development of ET research. Therefore, it is necessary to construct animal models. The structure of ET varies in size, structure, and tissue morphology across species. It is necessary to select the appropriate model animals. However, there is a lack of precise comparative studies on ET anatomical methods and tissue morphology across species. Therefore, it is difficult to select appropriate animal models for research.

The pathogenesis of ETD is unknown. At present, the clinical treatment of ETD is limited [6,7], and the treatment effect is poor. Therefore, the exploration of animal models of ET disease is helpful to further understand the pathogenesis of ETD [8,9]. Lipopolysaccharide (LPS) is an essential component of bacterial endotoxins and can activate macrophages and trigger the release of inflammatory cytokines in mammalian tissues. Recent studies have shown that intratympanic injection of LPS mimics acute otitis media (AOM) and can lead to morphological and functional changes in the inner ear [10,11]. However, the effects of intratympanic injection of LPS on the structure and function of ET remain unclear.

NADPH oxidase 2 (NOX2) is a key enzyme responsible for reactive oxygen species (ROS), acting as a pivotal mediator of oxidative stress, significantly influencing disease progression and treatment responses. The oxidative stress mediated by NOX2 plays a major role in the process of corneal injury and healing [12]. In Parkinson’s disease models, activation of NOX2 can mediate neuroinflammation, and inhibition of NOX2 can alleviate the neurotoxic effects associated with activated microglia [13]. This suggests that targeting NOX2 provides a compelling therapeutic strategy.

In this study, a combined skull base–nasal approach was designed for the dissection of ET tissue in mice, rats, and miniature pigs, which could completely remove ET tissue without the assistance of endoscopic instruments. Tissue staining was utilized to observe the differences in morphology and nerve fiber distribution of ET among species. To provide new ideas for the study of the pathogenesis of ETD from the perspective of comparative anatomy. At the same time, the expression changes in NOX2 in ET inflammation were explored. This serves as a reference for the pathological basis of ETD.

## 2. Methods

### 2.1. Animals and Anesthesia

Male healthy Bama miniature pigs (*n* = 3), aged 15–18 months, and weighing 25–30 kg were purchased from Peking University Third Clinical School of Medicine. Healthy, male Sprague-Dawley (SD) rats (*n* = 3), aged 6–8 weeks old, and weighing 200 g, and healthy, male C57BL/6J mice (*n* = 3), aged 6–8 weeks old, and weighing 20 g were purchased from the Peking University Health Science Center Department of Laboratory Animal Science. Animal experiments were reviewed and approved by the Experimental Animal Ethics Committee, Peking University Third Clinical School of Medicine (Date 21 November 2022, Approval No. SA2022576) and the Institutional Animal Care and Use Committee of Peking University Health Science Center (Date 26 March 2024, Approval No. DLASBD0161), and performed following the Chinese national guidelines for the care of laboratory animals. All efforts were made to minimize suffering.

Miniature pigs were given an intramuscular injection of Suta50+ (Virbac, Carros, France) cyrazine hydrochloride compound anesthetic. After the induction of anesthesia, tracheal intubation was performed, the ear vein was opened, and the respiratory anesthesia machine was set up with the following parameters: isoflurane, 3–3.5%; tidal volume, 12 mL/kg of body weight; and respiratory rate, 20 times/min. After entering a state of deep anesthesia (determined by a lack of response to forceps and syringe acupuncture on the abdominal skin), euthanasia was performed by rapid intravenous injection of 10% potassium chloride, and ECG monitoring was performed until the ECG waveform disappeared and the machine was withdrawn. Sprague-Dawley (SD) rats and C57BL/6J mice were deeply anesthetized with isoflurane and then sacrificed.

### 2.2. Anatomy

**Figure 1:**
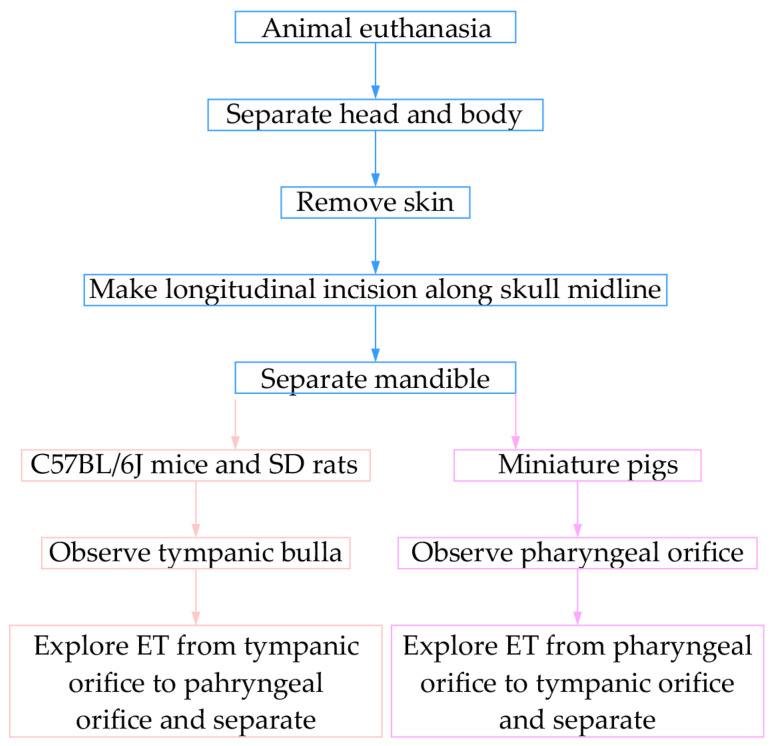
Anatomical sampling of ET via the combined cranial base–nasal approach.

The animal’s head and body were divided, the skin was removed, and then the mandible was separated. A longitudinal incision was made through the midline of the skull. At this point, tympanic bulla was observed in mice and rats. The ET was explored from the tympanic orifice to the pharynx orifice and separated; in miniature pigs, the pharyngeal orifice was observed in the nasal cavity, and the ET was divided from the pharyngeal orifice to the tympanic orifice (Figure 1).

### 2.3. Hematoxylin-Eosin Staining (H&E)

The obtained ET was kept in formalin. Then, nitric acid decalcification solution (Beyotime Biotech, Shanghai, China) was added for decalcification, and then the samples were dehydrated, transparent, and paraffin embedded. Continuous sections were at a thickness of 50 μm (pig), 10 μm (mouse). A hematoxylin-eosin (H&E) staining kit (Solarbio, Beijing, China) was used for tissue staining.

### 2.4. Luxol Fast Blue Myelin Staining

The paraffin sections of ET were dewaxed in water. To the wash, 95% ethanol was added. The ET was stained at room temperature for 20 h in Luxol Fast Blue dyeing solution (Leagene Biotechnology, Beijing, China). Next, 95% ethanol was added to wash away excess dyeing liquid, and then the sections were rinsed with distilled water. Differentiation was performed with Luxol differentiation solution. Then, 75% ethanol was added. After rinsing with distilled water, the sections were dehydrated conventionally, cleared with xylene, and mounted with neutral balsam.

### 2.5. Immunohistochemical Staining (IHC)

Immunohistochemistry was performed on 5 μm paraffin sections following standard procedures. After deparaffination and antigen retrieval in citric acid buffer, sections were blocked with 3% H_2_O_2_ and permeabilized with 5% goat serum and 0.5% Triton X-100. Primary antibodies against IL-1β (Cusabio, Wuhan, China, 1:1000), COX2 (Proteintech, Wuhan, China, 1:1000), and NOX2 (Abcam, Shanghai, China, 1:1000) were applied overnight at 4 °C, followed by incubation with a universal HRP-polymer secondary antibody (ZSGB-BIO, Beijing, China). Signal was developed using DAB (ZSGB-BIO, Beijing, China), counterstained with hematoxylin, dehydrated, and mounted for microscopic observation.

### 2.6. Inflammatory Model

Mice were provided with an intratympanic injection (IT) of LPS (Sigma, Shanghai, China, 2.5 mg/kg, 5 mg/mL) or 10 μL saline after anesthesia for 48 h. Then, the ET was dissected using a combined nasal–cranial base approach, and further tests were conducted.

### 2.7. Statistical Analysis

Statistical analysis was performed by GraphPad Prism 8 software (GraphPad Software, San Diego, CA, USA). Data were presented as the mean ± standard deviation (SD) and were analyzed with the two-tailed Student t-test or Brown-Forsythe and Welch’s ANOVA test. *p* value of <0.05 was represented statistically significant.

## 3. Results

### 3.1. Comparative Anatomical Approaches for ET Across Species

**Figure 2:**
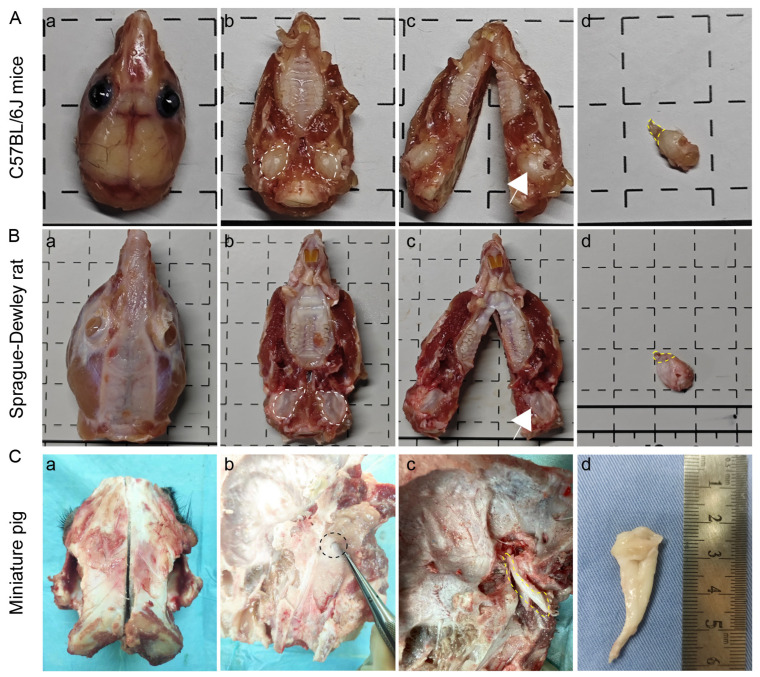
Anatomical sampling process of ET across species. (**A**,**B**) C57BL/6J mice and SD rats. (**a**) Isolated head and body. Remove the skin from the head and neck. (**b**) Removed mandible, exposing tympanic bulla and ET. The white dotted lines are the tympanic bulla and ET. (**c**) Separated skull along the midline. The white arrow indicates the tympanic bulla. (**d**) Displayed ET. The ET and tympanic bulla were completely separated. The yellow dashed line shows ET. (**C**) Miniature pigs. (**a**) Longitudinal section at the midline position of the skull. (**b**) Removal of the brain tissue and exposure of the inner ear bone structure. Black dashed line: Pharyngeal orifice of ET. (**c**) The inner ear structure was removed, and ET from the pharyngeal to the tympanic orifice was separated. Yellow dashed line: Location of ET. (**d**) Complete anatomy of ET.

Due to the distinct craniofacial anatomies of mice (Figure 2A), rats (Figure 2B), and miniature pigs (Figure 2C), species-specific ET dissection protocols were developed and compared. Compared with the miniature facial skull of rats and mice, the skulls of miniature pigs are thick and hard. The bony structure of the facial skull needs to be broken using a reciprocating saw, while the skull of rats and mice can be directly cut with scissors. In the exploration of the pharyngeal orifice and tympanic orifice of the ET, the tympanic orifice is obvious in miniature pigs. After removing the inner ear bone, the tympanic orifice could be observed by retrograde exploration along the ET pharyngeal orifice. However, the tympanic orifices of rats and mice are small and difficult to find, so the lateral wall of the pharynx needs to be pulled or probed from the tympanic orifice anterograde to the pharyngeal orifice.

### 3.2. There Is a Significant Difference in the Size of ET Across Species

The size of ET in C57BL/6J mice and SD rats was measured. The total length of ET in C57BL/6J mice is 1.05–1.28 (1.152 ± 0.084) mm. The maximum diameter of the pharyngeal orifice (PO) of ET is 0.10–0.14 (0.1133 ± 0.01633) mm, and the maximum diameter in the tympanic orifice (TO) is 0.17–0.38 (0.2283 ± 0.08085) mm. The total length of ET in SD rats is 3.68–3.80 (3.738 ± 0.04355) mm, the PO is 0.13–0.23 (0.18 ± 0.04) mm, and the TO is 0.41–0.47 (0.43 ± 0.0228) mm. The size of the ET in five ears from three miniature pigs was measured (one of the ETs was not completely removed). The total length of the ET in miniature pigs is 29.2–34.9 (32.340 ± 2.157) mm, and the PO of ET is 8.2–14.3 (10.60 ± 2.625) mm. The TO is 2.1–2.7 (2.34 ± 0.251) mm (Supplementary Table S1).

**Figure 3:**
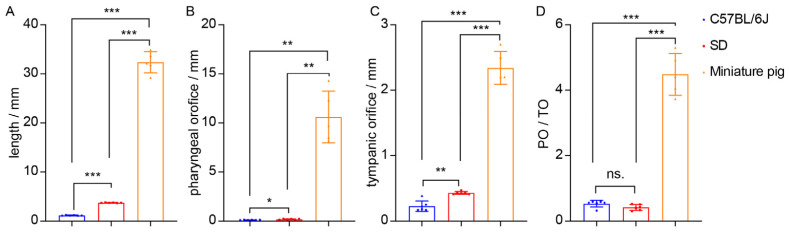
Comparison of ET sizes across species. (**A**–**C**) Length of ET. There are significant differences in the length, maximum diameters of ET pharyngeal opening (PO), and maximum diameters of ET tympanic opening (TO) among mice, rats, and miniature pigs. (**D**) Ratio of PO to TO (PO/TO). The PO/TO values of mice and rats are less than 1; the PO/TO values of miniature pigs are greater than 1. * *p* < 0.05, ** *p* < 0.01, *** *p* < 0.001, ns. no statistical difference.

Comparative analysis shows that ET length, PO, and TO in miniature pigs are larger than rats, and that in rats are larger than mice (Figure 3A–C). There is no significant difference in the PO/TO value between rats and mice, and both have a PO/TO value less than 1, suggesting that the pharyngeal orifice is narrower than the tympanic orifice in rodents. The PO/TO value in miniature pigs is significantly higher than that in mice and rats, and the PO/TO value in miniature pigs is greater than 1, indicating that the ET tympanic orifice in miniature pigs is narrower than the pharyngeal orifice (Figure 3D).

### 3.3. There Are Differences in the Tissue Morphology of ET Across Species

**Figure 4:**
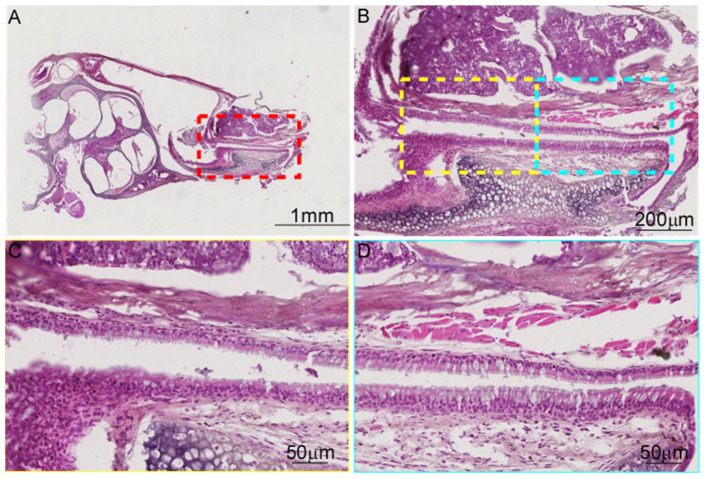
Morphology of ET in C57BL/6J mice. (**A**) Longitudinal section of ET and tympanic bulla under low magnification, ×1. (**B**) The cartilage exited in the whole process of ET, ×5. (**C**,**D**) ET in the high magnification, ×20.

**Figure 5:**
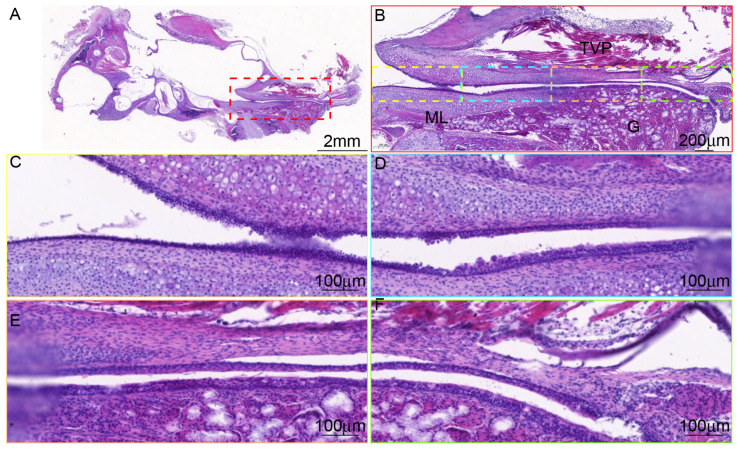
Morphology of ET in SD rats. (**A**) Longitudinal section of ET and tympanic bulla under low magnification, ×0.4. (**B**) The cartilage exited in the whole process of ET, ×5. (**C**–**F**) ET in the high magnification, ×10. ML, medial cartilage plate; TVP, Tensor veli palatini muscle; G, ET submucosal gland.

**Figure 6:**
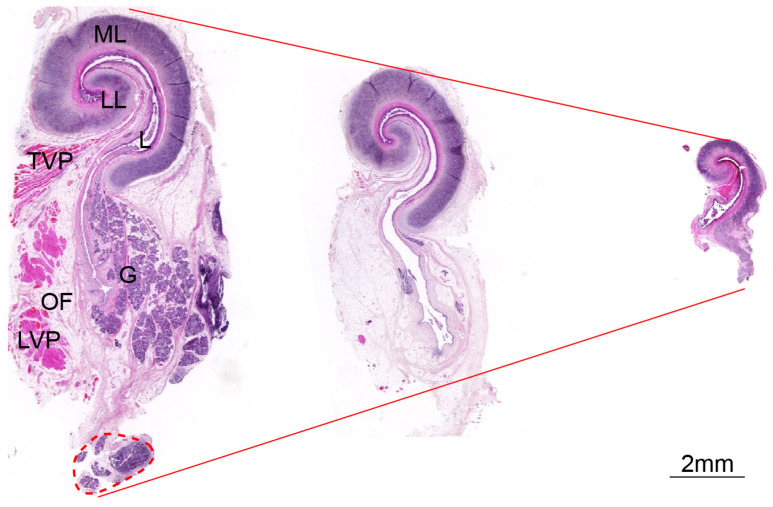
Cross-sectional structure of ET in pigs. G, ET submucosal gland; L, ET lumen; LL, lateral cartilage plate; ML, medial cartilage plate; OF, Ostmann’s fat pad; TVP, Tensor veli palatini muscle, LVP, Levator veli palatini muscle; The red dotted line represents the lymph node, ×0.4.

The epithelial cells at the tympanic orifice are relatively flat, gradually transitioning to a columnar morphology toward the pharyngeal orifice. Goblet cells are sparsely distributed near the tympanic orifice, whereas both goblet cells and ciliated cells are mainly distributed near the pharyngeal orifice in mice (Figure 4), similar to human ET [14,15]. The epithelial changes from the tympanic orifices to the pharyngeal orifices were not significant, and mucus glands were found near the pharyngeal orifice in rats (Figure 5). The ET is surrounded by cartilage (Figure 4, Figure 5 and Figure 6). The lateral cartilage plate is narrow, and the medial cartilage plate is wide in miniature pigs (Figure 5). The ET cartilage at the pharyngeal orifice is shaped like the number “9”, and gradually becomes shorter toward the tympanic orifice that is shaped like “C”. ET is surrounded by muscle and adipose tissue. Cut along the bottom of the ET mucosa. The ET mucosa is smooth, the cartilage at the pharyngeal ostium is trumpet-shaped, and the cartilage at the tympanic ostium is connected with the tympanic cavity in a “V” shape (Figure 6).

### 3.4. The Distribution of Myelinated Nerve Fibers in ET Across Species

**Figure 7:**
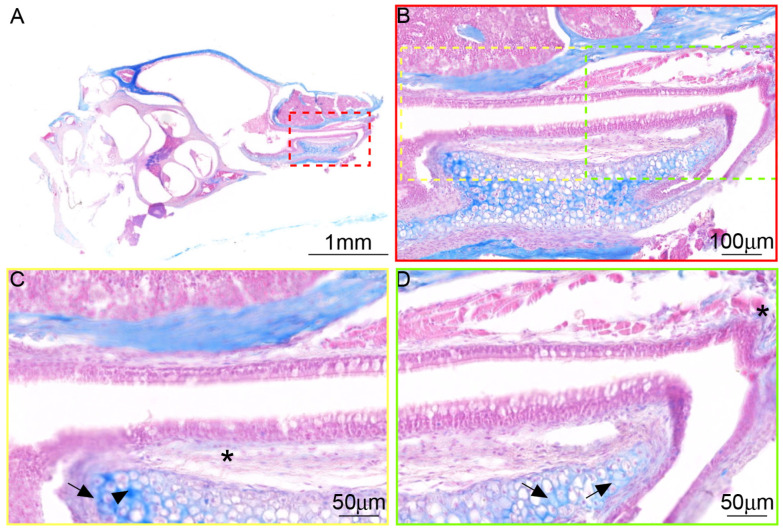
Myelin staining of C57BL/6J mice. (**A**) The distribution of nerves was observed at low magnification, ×1. (**B**) The nerves in ET were mainly distributed in cartilage, ×10. (**C**,**D**) A little intraepithelial myelin staining was observed in ET at high magnification, ×20. * Nerve in submucosal connective tissue, arrow: nerve longitudinal section, arrowhead: nerve transverse section.

**Figure 8:**
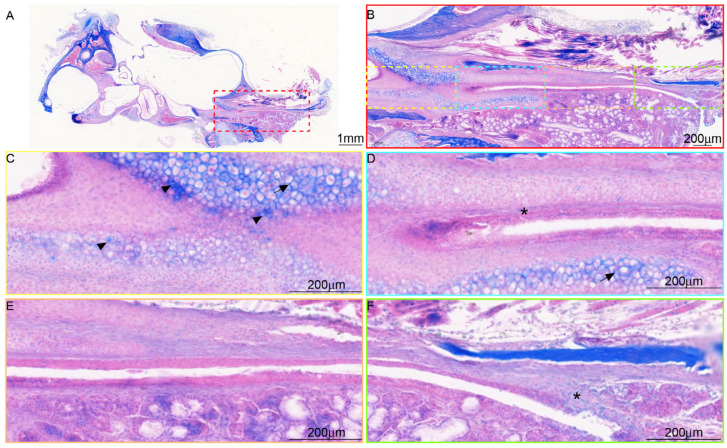
Myelin staining of SD rats. (**A**) The distribution of nerves in low magnification, ×1. (**B**) The nerves in ET were mainly distributed in cartilaginous, ×4. (**C**–**F**) The nerve of ET was mainly distributed in the cartilage tissue, and there were a few fiber branches in the submucosal tissue, ×20. * Nerve in submucosal connective tissue, arrow: nerve longitudinal section, arrowhead: nerve transverse section.

**Figure 9:**
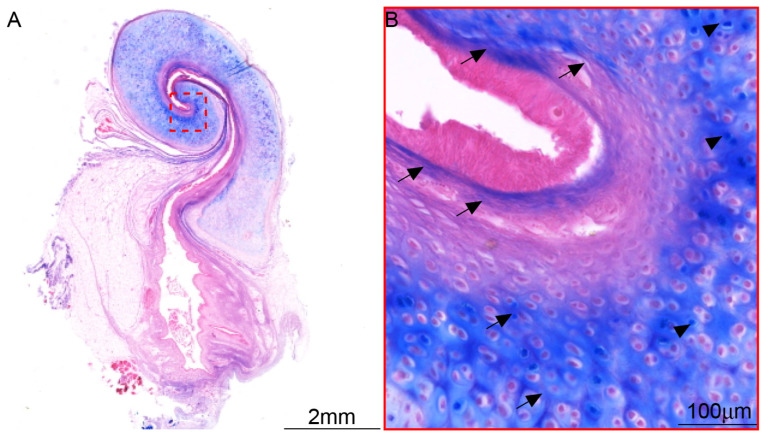
Myelin staining of miniature pigs. (**A**) Under a low magnifying microscope, the nerves of ET are mainly distributed in cartilage tissue and submucosal connective tissue, ×0.4. (**B**) The nerve at high magnification, ×10. Arrow: longitudinal section of nerve, arrowhead: nerve transverse section.

The nerve in ET is stained with Luxol fast blue solution in the paraffin sections, in C57BL/6J mice (Figure 7), SD rats (Figure 8), and miniature pigs (Figure 9). The comparative analysis revealed that in these three animals, the nerve fibers were mainly distributed in the ET cartilage and interwoven into a network. A small number of nerve fibers branched out in the ET submucosal tissue. The longitudinal section and transverse section of the nerve are visible. Nerves within the connective tissue of the ET lumen are aligned in the same direction as the collagen fibers, running parallel to the long axis of the lumen.

### 3.5. LPS Stimulation Can Induce ET Inflammation and the Expression of NOX2

**Figure 10:**
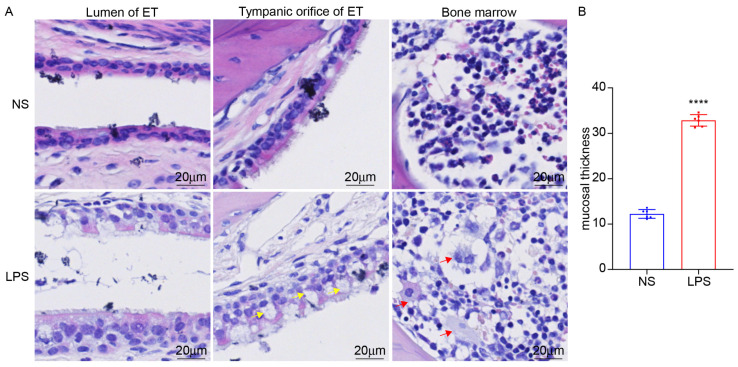
The ET inflammation model was constructed by LPS stimulation in C57BL/6J mice. (**A**,**B**) LPS stimulation caused mucosal thickening. Goblet cells in the tympanic orifice (yellow arrow) and inflammatory cells in the bone marrow around ET could be seen obviously in the LPS group (red arrow), ×40. **** *p* < 0.0001.

**Figure 11:**
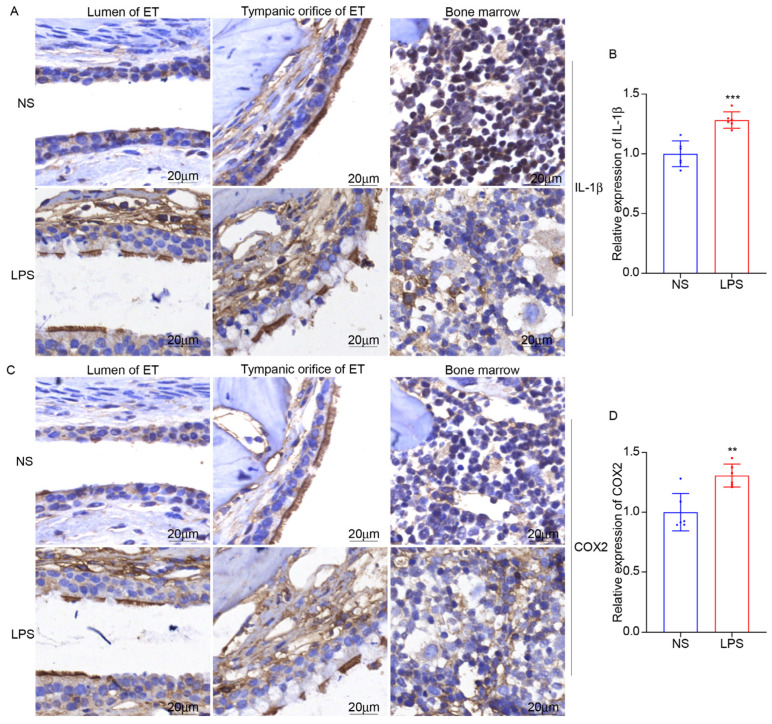
LPS promotes IL-1β and COX2 expression in ET, ×40. (**A**,**B**) The expression of IL-1β increased in ET in the LPS group. (**C**,**D**) The expression of COX2 increased in ET in the LPS group. ** *p* < 0.01, *** *p* < 0.001.

We established the mouse model of ET inflammation by intratympanic injection of LPS for 48 h. The mucosa of ET was swollen, and inflammatory cells increased (Figure 10). Inflammatory factors IL-1β and COX2 in ET and the surrounding bone marrow were significantly increased (Figure 11). Intratympanic LPS stimulation can effectively induce ET inflammation in mice, which is an effective animal model of otitis media in C57BL/6J mice.

**Figure 12:**
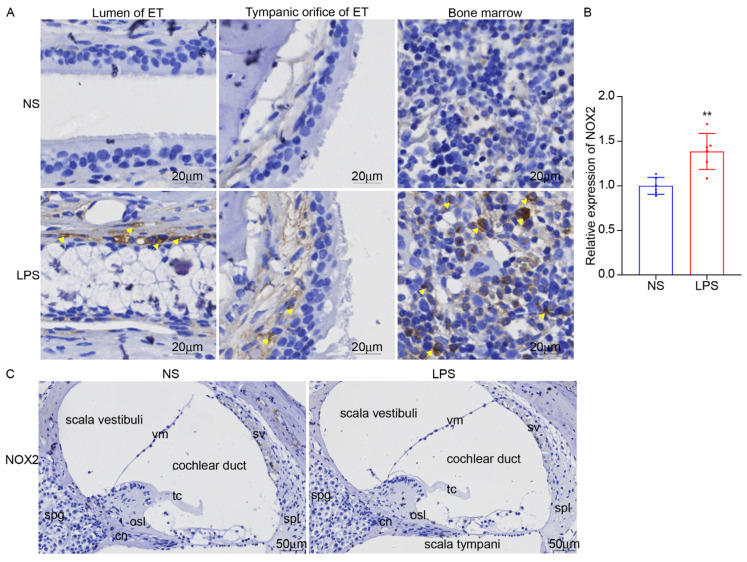
Immunohistochemical staining of NOX2 in ET. (**A**,**B**) The expression of NOX2 increased after LPS stimulation in ET (indicated by yellow arrows), ×40. (**C**) NOX2 was not increased after LPS stimulation in the inner ear, ×20. ** *p* < 0.01.

NADPH oxidase 2 (NOX2) plays a significant role in the generation of reactive oxygen species (ROS) in various physiological and pathological conditions. As a major source of ROS, NOX2 is involved in mediating oxidative stress and related damage in tissues. Inhibiting NOX2 can alleviate tissue damage in inflammatory disease models. NOX2 was significantly increased after LPS stimulation for 48 h in ET but not in the inner ear (Figure 12), suggesting that NOX2 has an important regulatory role in the inflammatory process of ET.

## 4. Discussion

The structure of ET shows significant interspecies differences across species, representing an adaptive selection of species to ecological pressures and physiological needs [8]. Rodents ET (mice/rats) are mainly of cartilage structure and have smaller pharyngeal openings. ET length in mice is 1.152 ± 0.084 mm, and the PO/TO is 0.5283 ± 0.1034. In rats, ET length is 3.738 ± 0.04355 mm, and the PO/TO is 0.4167 ± 0.08869, similar to mice. While in miniature pigs, ET length in mice is 32.340 ± 2.15700 mm with a smaller tympanic orifice, and the PO/TO is 4.482 ± 0.6361. Rodents (mice/rats) have obvious tympanic bulla structures, which are significantly different from miniature pigs. This may relate to the physiological need to fit rapid middle ear pressure regulation due to their cave-dwelling lifestyle. The short and narrow ET lumen and PO/TO ratio are not conducive to the rapid flow of air. To achieve a rapid balance of middle ear pressure, the tympanic bulla of rodents expands the middle ear volume, which is beneficial for maintaining middle ear pressure. The ET cartilage in miniature pigs presents a gradually conical structure towards the pharyngeal orifice, with a relatively close length to human ET [16].

The cartilaginous portion plays a critical role in the physiological function of ET by enabling its opening and closing [17]. The “9-shaped” cartilage plate of the miniature pig (Figure 6) is slightly different from the “C-shaped” of humans, but the functional regulatory structures, such as the tensor veli palatini muscle (TVP), levator veli palatini muscle (LVP), submucosal glands, and nerve distribution, are similar. The cartilage forms the top and inner, and outer walls of ET. The medial cartilage plate is wider compared to the lateral cartilage plate. The opening and closing of ET are regulated by TVP and LVP. Submucosal glands and mucosal folds can be seen in the middle and lower parts of the lumen [18]. The similarity mainly stems from the fact that both pigs and humans are terrestrial omnivores. They need to maintain the balance of middle ear pressure through frequent chewing and swallowing. In addition, the distribution of myelinated nerve fibers in ET is similar across species. The nerve fibers are mainly distributed in the cartilage. A small number of nerve fiber branches are distributed in the submucosal, suggesting that the nerves may regulate the activity of cartilage to maintain the opening and closing of ET. And we have summarized the ET structure across species (Supplementary Table S2).

ET morphological differences across species may determine the selection of model animals. ET animal models mainly originate from small and medium-sized animals such as rats, guinea pigs, and rabbits. They have similarities in morphology and structure with the human ET, but the structures are much smaller than humans. In recent years, scholars have discovered that the pig ET is similar in size and structure to the human one, which can better simulate ET cartilage [8]. We found that the mice/rats ET was more similar to the human ET bone part, which is similar to the human ET isthmus bone and cartilage, as well as the gradual narrowing of the tube cavity from the tympanic orifice to the isthmus. In addition, mice/rats ET mucosal epithelial cells were also similar to those of human ET. Goblet cells were mainly distributed in the pharyngeal orifice with dense cilia. The pig ET was more similar to the human ET cartilage part. Therefore, we hypothesized that the mice/rat might be a nice choice for studies related to bone or mucosa-related ETD, while the pig model might be suitable for research about cartilage-related ETD. Currently, the pig model is mainly used in the evaluation of balloon dilation [19]. Rodent animals are mainly used for studies related to the cleaning and protective functions of ET [20,21].

In our study, LPS was injected into mice ET to construct an ET inflammation model and explore the key molecules involved in ET inflammation. We found that NOX2 was elevated by about 38.6% after LPS stimulation, and led to ET IL-1β, and COX2 being elevated about 28.4% and 30.8%. NOX2 is a member of the NADPH oxidase family and participates in various pathological physiological processes by catalyzing the oxidation of NADPH to generate superoxide O^2−^. In macrophages, NOX2 is highly expressed and participates in immune regulation by catalyzing the NOX2/ROS pathway [22]. NOX2 can also promote the release of inflammatory factors by activating the MAPK signaling pathway [23,24]. NOX2 inhibition can reduce neural inflammation and maintain neuronal integrity [25,26]. A recent study found that NOX2 is a master regulatory molecule for the fragility of outer hair cells (OHC) in the cochlea of patients with frequent hearing loss. After NOX2 knockout, oxidative stress damage was significantly reduced. In this study, the expression of NOX2 increased in ET (Figure 12A,B), but it did not induce NOX2 expression in the inner ear (Figure 12C), suggesting that short-term LPS stimulation mainly causes NOX2-mediated oxidative stress damage to ET, but does not cause NOX2-related inner ear damage. Whether the long-term stimulation with LPS could lead to the expression of NOX2 in the inner ear remains to be explored further. Furthermore, this section mainly focuses on the morphological changes in ET. We will further explore the influence of LPS on ET function regulation in the future.

## 5. Conclusions

The combined skull base–nasal approach is an effective method to anatomy ET across species. Rodent species (mouse: 1.152 ± 0.084 mm, rat: 3.738 ± 0.04355 mm) tympanic bulla structures are useful for maintaining middle ear pressure. Miniature pig ET cartilage (32.34 ± 2.157 mm) gradually expands towards the pharyngeal orifice, which is similar to human cartilage structure. NOX2-mediated oxidative stress can promote the release of inflammatory factors and thicken the ET mucosa. This study provides evidence for ET sampling from different species and provides reference suggestions for the selection of model animals for different research purposes. Moreover, further exploration clarifying the key role of NOX2 in ETD is expected to provide new targets for ETD treatment and promote the advancement of ETD treatment from surgical treatment to molecular treatment.

## Data Availability

The original data presented in this study are available on reasonable request from the corresponding author. The data are not publicly available due to privacy concerns.

## Supplementary Materials

The following supporting information can be downloaded at: https://www.mdpi.com/article/10.3390/audiolres15050141/s1, Table S1: Size measurement of ET in miniature pigs, mice and rats; Table S2: Comparison of ET Structure across Species.
