# Supplementary material for: Comparative Anatomical and Morphometric Analysis of Eustachian Tube Across Species
Source: Audiol Res. 2025 Oct 21;15(5):141. doi: 10.3390/audiolres15050141 (PMC12561518; doi:10.3390/audiolres15050141)
Supplement: Supplementary file 1 [file audiolres-15-00141-s001.zip › audiolres-3828821-supplementary.pdf]

Supplementary Table S1. Size measurement of ET in miniature pigs, mice and rats.

Animal	Number	Length/ mm	Mean ± SD/mm	PO/mm	Mean ± SD/mm	TO/mm	Mean ± SD/mm
Miniature pigs	1	33.7	32.34 ± 2.157	14.3	10.6 ± 2.625	2.70	2.34 ± 0.251
	2	34.9		12.3		2.50	
	3	31.8		8.20		2.20	
	4	29.2		8.50		2.10	
	5	32.1		9.70		2.20	
C57BL/6J mice	1	1.22	1.152 ± 0.084	0.12	0.1133 ± 0.01633	0.38	0.2283 ± 0.08085
	2	1.28		0.14		0.25	
	3	1.10		0.10		0.17	
	4	1.14		0.12		0.22	
	5	1.05		0.10		0.17	
	6	1.12		0.10		0.18	
Sprague-Dawley (SD) rats	1	3.71	3.738 ± 0.04355	0.13	0.18 ± 0.04	0.43	0.43 ± 0.0228
	2	3.75		0.20		0.47	
	3	3.72		0.21		0.41	
	4	3.68		0.14		0.41	
	5	3.77		0.23		0.44	
	6	3.80		0.17		0.42	

Supplementary Table S2. Comparison of ET Structure across Species

Animals	Slicing	Bone	Cartilage	TVP	LVP	Lymph node	Goblet cells	Gland
C57BL/6J mice	Longitudinal section	Cover cartilage	ET full length	Lateral cartilage plate	Parallel to lumen	/	Mainly in pharyngeal orifice	mixed acinus
Sprague-Dawley (SD) rats	Longitudinal section	Cover cartilage	ET full length	Lateral cartilage plate	Parallel to lumen	/	Mainly in pharyngeal orifice	mixed acinus
Miniature pigs	Transverse section	/	"9" shape	Lateral cartilage plate	Parallel to lumen	√	Not prominent.	mixed acinus

Note: /, Not observed; √, existed.
